# Sensorless Adaptive Voltage Control for Classical DC-DC Converters Feeding Unknown Loads: A Generalized PI Passivity-Based Approach

**DOI:** 10.3390/s21196367

**Published:** 2021-09-24

**Authors:** Walter Gil-González, Oscar Danilo Montoya, Carlos Restrepo, Jesus C. Hernández

**Affiliations:** 1 Facultad de Ingeniería, Institución Universitaria Pascual Bravo, Campus Robledo, Medellín 050036, Colombia; walter.gil@pascualbravo.edu.co; 2 Department of Electrical Engineering, University of Jaén, Campus Lagunillas s/n, Edificio A3, 23071 Jaén, Spain; 3 Facultad de Ingeniería, Universidad Distrital Francisco José de Caldas, Bogotá 110231, Colombia; odmontoyag@udistrital.edu.co; 4 Laboratorio Inteligente de Energía, Universidad Tecnológica de Bolívar, Cartagena 131001, Colombia; 5 Department of Electromechanics and Energy Conversion, Universidad de Talca, Curicó 3340000, Chile

**Keywords:** generalized passivity-based controller, second-order DC-DC converters, averaging model in converters, port-controlled hamiltonian systems

## Abstract

The problem of voltage regulation in unknown constant resistive loads is addressed in this paper from the nonlinear control point of view for second-order DC-DC converters. The converters’ topologies analyzed are: (i) buck converter, (ii) boost converter, (iii) buck-boost converter, and (iv) non-inverting buck-boost converter. The averaging modeling method is used to model these converters, representing all these converter topologies with a generalized port-Controlled Hamiltonian (PCH) representation. The PCH representation shows that the second-order DC-DC converters exhibit a general bilinear structure which permits to design of a passivity-based controller with PI actions that ensures the asymptotic stability in the sense of Lyapunov. A linear estimator based on an integral estimator that allows reducing the number of current sensors required in the control implementation stage is used to determine the value of the unknown resistive load. The main advantage of this load estimator is that it ensures exponential convergence to the estimated variable. Numerical simulations and experimental validations show that the PI passivity-based control allows voltage regulation with first-order behavior, while the classical PI controller produces oscillations in the controlled variable, significantly when the load varies.

## 1. Introduction

Nowadays, the electrical distribution networks also include direct current (DC) network operation with low- and high-voltage applications. This has been caused for two main reasons; the first of them is the growing penetration of renewable energy sources (e.g., solar and wind energy), energy storage systems (e.g., battery, supercapacitor storage, super magic storage, and among), and controllable loads, under the idea of DC-microgrids or DC-distribution [1]. The second reason is the application of medium-voltage DC grids offshore wind power applications [2]. Furthermore, the DC grids are more efficient and easier to operate than the alternating current networks since they should only be interested in controlling the active power and regulating the node DC voltages, while the concepts such as control of the reactive power or frequency disappear [3,4]. Hence, the DC grids can have lower power losses and better voltage profiles than the alternating current networks [4].

New technologies such as renewable energy sources and energy storage systems use power electronic converters to be integrated into DC grids [5]. The converters can control the variables (voltage or power outputs) of these technologies; for this reason, the study of the control strategies of DC converters has become an essential focus. These strategies manage all the state variables of the DC grids, permitting the execution of the primary and secondary control stages [6]. This indicates that the power electronic converters and their controls are important to the operation of DC grids.

Different converter types can be installed for the operation of the DC grids depending on the requirements of the network. The DC-DC converters typical are buck, boost, buck-boost, and non-inverting buck-boost converters [7]. The buck and boost converter can be implemented in the integration of wind and solar photovoltaic sources since in these applications, the energy flow always goes from renewable source to the DC grid [8,9]. Meanwhile, buck-boost converters are usually implemented to the battery interface and supercapacitor devices, which have energy flow in both senses. This indicates that in some periods, the energy flow goes from the DC grid to these devices in order to charge them, and they can supply energy to the DC grid in other periods [10].

In the current literature regarding control techniques applied for second-order DC-DC converters for output voltage regulation, the following can be found: sliding-mode controllers [11,12], fractional-order sliding mode control [13], nonlinear high-gain observer-based second-order sliding mode control [14], observer-based higher-order sliding mode control [15], backstepping control designs [16,17], exact feedback linearization methods [18,19], adaptive control strategies [20], passivity-based control designs [7,21], and linear methods such as PI or feedback designs [22], among others. The passivity-based controller has already been implemented for these types of converters; however, its analysis has been carried out separately, not allowing a generalized control law design. Therefore, this study proposes a general port-Controlled Hamiltonian modeling for these topologies that includes simulation and experimental validations with a unified (i.e., general) PI-PBC control law. The main advantage of having a general control design for the most classical DC-DC converters topologies lies in the fact that the modern electrical networks operated in with the DC technologies involves most of these converters in different isolated or connected grid applications [23]. Some of these applications are battery chargers [24,25], photovoltaic generation [26,27], and voltage-controlled loads [28,29], among others, which implies that a generalized control strategy can be used indifferently of the application ensuring stability during closed-loop operation. One of the most important features of our proposed PI-PBC controller is the possibility of taking advantage of the PI gains to eliminate steady-state errors with the security that the closed-loop dynamical system will be asymptotically stable in the sense of Lyapunov, which is a characteristic that can be complex to prove in classical PI designs.

An additional characteristic of the proposed PI-PBC controller is regarding the required sensors to implement the controller. To reduce the number of current sensors, we employ a linear integral load estimator that ensures exponential convergence to the unknown resistive load value, making our proposed PI-PBC design an adaptive controller with a reduced number of sensors. This control approach shows excellent numerical performance compared with classical PI designs in both simulated and experimental cases.

The remainder of this research is structure as follows: Section 2 summarizes the main aspects of the passivity-based control theory with PI actions for bilinear dynamical systems. Section 3 presents the derivation of the average model for the four DC-DC converter topologies analyzed in this research, including the unified general model and the application of the PI-PBC to regulated the output voltage. Section 4 presents the design of the linear integral estimator to determine the value of the unknown resistive load that helps with the reduction of a current sensor in the load terminals. Section 5 presents all the simulations and experimental validations in all the converter topologies with their corresponding analyses and discussions. Finally, the main concluding remarks are presented in Section 6.

## 2. Generalized PI Passivity-Based Controller Design

The passivity-based control design is a robust and well-known nonlinear control theory that exploits the general Hamiltonian representation for a class of dynamical systems [30,31], that can be represented as follows:

(1) $$\begin{array}{c} \dot{x}=\left[J(x,u)-R(x)\right]\frac{\partial H(x)}{\partial x}+\xi , \end{array}$$

where *x* is the vector of states, J(x,u) is a skew-symmetric matrix that depends on the states and control inputs (*u*), R(x) is a positive semidefinite matrix that can depend on the states, H(x) is the Hamiltonian energy storage function, and ξ represents a vector with external inputs.

Considering the Hamiltonian function H(x), the passivity-based control theory can design nonlinear controllers that ensure asymptotic convergence to the desired references; the PBC and the PI-PBC interconnection and damping assignment designs are the most applied methodologies in the field of the power converter applications [32,33,34]. Here, we propose applying the PI-PBC design for the second-order DC-DC converters since they exhibit a bilinear structure when connected to linear resistive loads (ideal for PI-PBC designs), which allows for exploiting the well-known advantages of the PI actions, with the main advantage that the stability operation in closed-loops is ensured [34].

### 2.1. The Bilinear System and the Incremental Model

The passivity-based control has different strategies to deal with a subclass of nonlinear systems called “bilinear systems” since their structure allows designing a PI passivity-based controller that ensures asymptotically stability in the sense of Lyapunov for closed-loop operation [34]. The general dynamical structure of a bilinear system in Hamiltonian form is defined in (2).

(2) $$\begin{array}{c} Q\dot{x}=\left(J_{0}+J_{1}u-R\right)x+bu+\xi , \end{array}$$

where $x\in R^{n\times 1}$ and $\xi \in R^{n\times 1}$ define the vector of state variables and external inputs; $u\in R^{m\times 1}$ represents the vector of control signals; $Q=Q^{T}≻0\in R^{n\times n}$ is a positive definite matrix that contains the parameters related to the elements that store energy; $J_{0,1}=-J_{0,1}^{T}\in R^{n\times n}$ are known as the interconnection matrices with the main characteristic that these exhibit a skew-symmetry structure; $R\in R^{n\times n}$ corresponds to the dissipation matrix; and $b\in R^{n\times m}$ represents the matrix that relates all the states with the inputs, which is simply known as the input matrix.

From the bilinear dynamical system (2) it is possible to achieve an incremental model that allows for designing the PI passivity-based controller on a new system of coordinates [34]. For this purpose, let us define the incremental variables $\tilde{x}$ and $\tilde{u}$ as follows: $\tilde{x}=x-x^{⋆}$ and $\tilde{u}=u-u^{⋆}$, where $x^{⋆}$ is the assignable equilibrium point to the bilinear system (2). Note that at equilibrium, the following relation is satisfied:

(3) $$\begin{array}{c} Q{\dot{x}}^{⋆}=\left(J_{0}+J_{1}u^{⋆}-R\right)x^{⋆}+bu^{⋆}+\xi . \end{array}$$

Note that if Equation (3) is subtracted from (2), the following incremental model is obtained:

(4) $$\begin{array}{c} Q\dot{\tilde{x}}=\left(J_{0}-R\right)\tilde{x}+J_{1}\left(ux-u^{⋆}x^{⋆}\right)+b\tilde{u}, \end{array}$$

Now, adding and subtracting the term $J_{1}ux^{⋆}$ in (4) and defining $g\left(x^{⋆}\right)=J_{1}x^{⋆}+b$, the following incremental model can be rewritten as follows:

(5) $$\begin{array}{c} Q\dot{\tilde{x}}=\left(J_{0}+J_{1}u-R\right)\tilde{x}+g\left(x^{⋆}\right)\tilde{u}. \end{array}$$

The most important characteristic of the incremental model (5) is that it is passive from the control signal $\tilde{u}$ to the output $\tilde{y}$, when the following energy storage function $H:R^{n}⟶R$ is considered:

(6) $$\begin{array}{c} H\left(\tilde{x}\right)=\frac{1}{2}{\tilde{x}}^{T}Q\tilde{x}, \end{array}$$

To demonstrate that the incremental dynamic model is passive, let us take the time derivative of the storage function as follows:

(7) $$\begin{array}{cc} \dot{H}\left(\tilde{x}\right) & ={\tilde{x}}^{T}Q\dot{\tilde{x}}\\ \; & ={\tilde{x}}^{T}\left(\left(J_{0}+J_{1}u-R\right)\tilde{x}+g\left(x^{⋆}\right)\tilde{u}\right),\\ \; & ={\tilde{x}}^{T}\left(J_{0}+J_{1}u-R\right)\tilde{x}+{\tilde{x}}^{T}g\left(x^{⋆}\right)\tilde{u},\\ \; & =-{\tilde{x}}^{T}R\tilde{x}+{\tilde{x}}^{T}g\left(x^{⋆}\right)\tilde{u}, \end{array}$$

(8) $$\begin{array}{cc} \dot{H}\left(\tilde{x}\right) & \leq {\tilde{x}}^{T}g\left(x^{⋆}\right)\tilde{u}, \end{array}$$

Now, if the output is defined as $\tilde{y}=g{\left(x^{⋆}\right)}^{T}\tilde{x}$, then, the following inequality holds:

(9) $$\begin{array}{c} \dot{H}\left(\tilde{x}\right)\leq {\tilde{u}}^{T}\tilde{y},\;\forall t\geq 0, \end{array}$$

which confirms that for any input $\tilde{u}$, the incremental model (5) is indeed passive.

### 2.2. Controller Design

The passive nature of the incremental model (5) observed from the control input $\tilde{u}$ to the output $\tilde{y}$ makes possible to design a PI controller that ensures stability properties in the sense of Lyapunov for closed-loop operation. For this purpose, let us define the following PI control structure:

(10) $$\begin{array}{cc} \tilde{u} & =-K_{p}\tilde{y}+K_{i}z, \end{array}$$

(11) $$\begin{array}{cc} \tilde{z} & =-\tilde{y}, \end{array}$$

where $K_{p}>0$, $K_{i}>0$, and $z\in R^{m\times 1}$ are the proportional and integral control gains, and *z* is a vector of auxiliary variables that helps with controller design.

To demonstrate that the PI passivity-based controller defined in (10) and (11) is stable, we consider the following Lyapunov candidate function:

(12) $$\begin{array}{c} V\left(\tilde{x},z\right)=H\left(\tilde{x}\right)+\frac{1}{2}z^{T}K_{i}z, \end{array}$$

which has the time derivative along the trajectories defined in (5) presented below:

(13) $$\begin{array}{cc} \dot{V}\left(\tilde{x},z\right) & =\dot{H}\left(\tilde{x}\right)+z^{T}K_{i}\dot{z},\\ \; & =-{\tilde{x}}^{T}R\tilde{x}+{\tilde{y}}^{T}\tilde{u}-z^{T}K_{i}\tilde{y},\\ \; & =-{\tilde{x}}^{T}R\tilde{x}+{\tilde{y}}^{T}\left(-K_{p}\tilde{y}+K_{i}z\right)-z^{T}K_{i}\tilde{y},\\ \; & =-{\tilde{x}}^{T}R\tilde{x}-{\tilde{y}}^{T}K_{p}\tilde{y}\leq 0, \end{array}$$

which shows that the equilibrium point $\tilde{x}=0$ is stable in the sense of Lyapunov with asymptotically properties as demonstrated in [35].

### 2.3. Assignable Equilibrium Point

The assignable equilibrium point for a bilinear dynamical system corresponds to the point where the system goes in a steady-state condition, i.e., $x^{⋆}$. Note that this point is univocally defined for the system (2) if this corresponds to a set of constant references [7]. Note that from (3) if $x^{⋆}$ is constant, then the following relation is obtained:

(14) $$\begin{array}{c} 0=\left(J_{0}-R\right)x^{⋆}+g\left(x^{⋆}\right)u^{⋆}+\xi , \end{array}$$

which is fulfilled for some constant control input $u^{⋆}$. Note that this control input can be obtained from (14) as follows:

(15) $$\begin{array}{c} u^{⋆}=-{\left(g{\left(x^{⋆}\right)}^{T}g\left(x^{⋆}\right)\right)}^{-1}g{\left(x^{⋆}\right)}^{T}\left(\left(J_{0}-R\right)x^{⋆}+\xi \right), \end{array}$$

which is satisfied if and only if ${\left(g{\left(x^{⋆}\right)}^{T}g\left(x^{⋆}\right)\right)}^{-1}$ is a full-rank matrix [7].

On the other hand, if we define the full-rank left-annihilator of $g\left(x^{⋆}\right)$ as $G\left(x^{⋆}\right)$, then, we know that $G\left(x^{⋆}\right)g\left(x^{⋆}\right)=0$ [36]. With this definition, it is possible to obtain the non-controlled variable from (14) using the following general relation.

(16) $$\begin{array}{c} G\left(x^{⋆}\right)\left(\left(J_{0}-R\right)x^{⋆}+\xi \right)=0. \end{array}$$

## 3. General Converters’ Modeling

**Figure 1:**
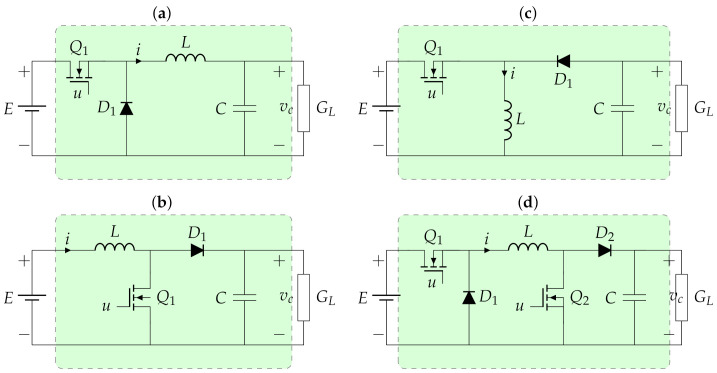
Second-order DC-DC converters: (**a**) buck converter, (**b**) boost converter, (**c**) buck-boost converter, (**d**) non-inverting buck-boost converter.

This section presents the general dynamic models for the most classical DC-DC converters, such as the buck, boost, buck-boost, and non-inverting buck-boost topologies. The main characteristic of these converters’ groups is that these are classified as second-order converters since each one of them includes two dynamics associated with their inductors and capacitors [7]. Figure 1 presents the general structure of the studied converters, where it is observed that these are connected to constant resistive loads, which is modeled as a conductance, i.e., $G_{L}$.

The variables and parameters in Figure 1 have the following interpretation: E>0 corresponds to the input voltage, i>0 represents the current that flows through the inductor *L*, $v_{c}>0$ represents the variable associated with the voltage output at the terminals of the capacitor *C*, and u∈[0,1] represents the control input applied to the forced-commutated switches. To have a consistent formulation for each one of the converters, we redefine the state variables $x_{1}=i$, and $x_{2}=v_{c}$. Each one of the dynamic models for the converters depicted in Figure 1 is described below.

### 3.1. Dynamic Model of the Buck Converter

The buck converter depicted in Figure 1a is widely known as the step-down converter since the output voltage is a fraction of the input voltage [37]. However, the reduction of the voltage input implies that the load current is higher when compared with the input. This type of converter is mainly used for regulating voltage in variable loads [7].

To obtain the average dynamic model of the buck converter, Kirchhoff’s laws are applied to the trajectory that contains the inductor and the node that connects the capacitor in Figure 1a. These laws produce the following dynamic model.

(17) $$\begin{array}{c} L{\dot{x}}_{1}=-x_{2}+uE, \end{array}$$

(18) $$\begin{array}{c} C{\dot{x}}_{2}=x_{1}-G_{L}x_{2}. \end{array}$$

Note that the main characteristic of the buck model is that it exhibits a linear relationship between the state variables $x_{1}$ and $x_{2}$ and the control input *u*, which corresponds to the duty cycle bounded between 0 and 1.

### 3.2. Dynamic Model of the Boost Converter

The boost converter in Figure 1b allows controlling the voltage output with magnitudes superior to the voltage concerning the input voltage [38]. This implies that the magnitude of the load current is inferior to the current flowing through the converter inductor [7]. The average dynamic model for the boost converter depicted in Figure 1b takes the following form.

(19) $$\begin{array}{c} L{\dot{x}}_{1}=-\left(1-u\right)x_{2}+E, \end{array}$$

(20) $$\begin{array}{c} C{\dot{x}}_{2}=\left(1-u\right)x_{1}-G_{L}x_{2}. \end{array}$$

### 3.3. Dynamic Model of the Buck-Boost Converter

The buck-boost converter in Figure 1c allows controlling the voltage output in a range that includes higher or lower voltage for the input voltage [38]. However, the main characteristic of this converter is that the output voltage has the opposite polarity concerning the input signal [7]. The average dynamic model for the boost converter depicted in Figure 1c takes the following form.

(21) $$\begin{array}{c} L{\dot{x}}_{1}=\left(1-u\right)x_{2}+uE, \end{array}$$

(22) $$\begin{array}{c} C{\dot{x}}_{2}=-\left(1-u\right)x_{1}-G_{L}x_{2}. \end{array}$$

### 3.4. Dynamic Model of the Non-Inverting Buck-Boost Converter

The non-inverting buck-boost converter in Figure 1d works similarly to the buck-boost topology, i.e., the output voltage can be higher or lower than the voltage input [22]. However, this converter maintains the same polarity of the voltage input [7]. The average dynamic model for the non-inverting buck-boost converter depicted in Figure 1c takes the following form.

(23) $$\begin{array}{c} L{\dot{x}}_{1}=-\left(1-u\right)x_{2}+uE, \end{array}$$

(24) $$\begin{array}{c} C{\dot{x}}_{2}=\left(1-u\right)x_{1}-G_{L}x_{2}. \end{array}$$

The difference of this converter concerning the three previous topologies is that this uses two forced commutated switches, while the buck, boost, and buck-boost converters only use one controlled switch. However, when the dynamic model of the non-inverting buck-boost converter is compared with the classical buck-boost converter, this only changes in the signs of the factors (1−u) responsible for the absolute polarity of the output voltage.

### 3.5. General Bilinear Representation of the Converters

The average dynamic models defined from (17) to (24) for the four studied second-order converter topologies can be generalized with a unique bilinear representation using an α-coefficient to select each one of the configurations [7]. The general bilinear model for these converters is defined below:

(25) $$\begin{array}{c} Q\dot{x}=\left(\alpha_{1}J_{0}-R\right)x+g\left(x\right)u+\alpha_{4}\xi , \end{array}$$

where $g\left(x\right)=\alpha_{2}J_{1}x+\alpha_{3}b$, and

(26) $$\begin{array}{c} Q=\left[\begin{array}{cc} L & 0\\ 0 & C \end{array}\right],\;J_{0}=\left[\begin{array}{cc} 0 & -1\\ 1 & 0 \end{array}\right],\;J_{1}=\left[\begin{array}{cc} 0 & 1\\ -1 & 0 \end{array}\right],\;R=\left[\begin{array}{cc} 0 & 0\\ 0 & G_{L} \end{array}\right],\;b=\left[\begin{array}{c} E\\ 0 \end{array}\right],\;\xi =\left[\begin{array}{c} E\\ 0 \end{array}\right]. \end{array}$$

Note that the α-coefficients for each one of the converters are reported in Table 1.

### 3.6. General Controller Structure

To design the controller for the general bilinear representation of the four DC-DC converters defined by (26), we consider that the complete general control law is

(27) $$\begin{array}{cc} u & =\tilde{u}+u^{⋆}=-K_{p}\tilde{y}+K_{i}z+u^{⋆}, \end{array}$$

(28) $$\begin{array}{cc} \dot{z} & =-\tilde{y}, \end{array}$$

which implies that we need to determine the value of the desired control input $u^{⋆}$ and the passive output $\tilde{y}$.

To determine the desired control input, it is used (15) in the general bilinear system (25), which produces:

(29) $$\begin{array}{c} u^{⋆}=-\frac{\left(\alpha_{3}E+\alpha_{2}x_{2}^{⋆}\right)\left(\alpha_{4}E-\alpha_{1}x_{2}^{⋆}\right)-\alpha_{2}x_{1}^{⋆}\left(\alpha_{1}x_{1}^{⋆}-G_{L}x_{2}^{⋆}\right)}{{\left(\alpha_{3}E+\alpha_{2}x_{2}^{⋆}\right)}^{2}+{\left(\alpha_{2}x_{1}^{⋆}\right)}^{2}}. \end{array}$$

Note that the desired control input in (29) depends on the value of the non-controlled variable $x_{1}^{⋆}$, which must be calculated to make the implementation of the proposed PI passivity-based controller. For this purpose, let us define the following left annihilator:

(30) $$\begin{array}{c} G\left(x\right)=\left[\begin{array}{cc} \alpha_{2}x_{1}^{⋆} & \alpha_{2}x_{2}^{⋆}+\alpha_{3}E \end{array}\right], \end{array}$$

which, combined with (16), allows for calculating the general expression for the non-controlled variable $x_{1}^{⋆}$ as follows:

(31) $$\begin{array}{c} x_{1}^{⋆}=\frac{G_{L}x_{2}^{⋆}\left(\alpha_{3}E+\alpha_{2}x_{2}^{⋆}\right)}{\alpha_{1}\left(\alpha_{3}E+\alpha_{2}x_{2}^{⋆}\right)+\alpha_{2}\left(\alpha_{4}E-\alpha_{1}x_{2}^{⋆}\right)}, \end{array}$$

where the main restriction is that the reference value for the controlled variable $x_{2}^{⋆}$ must be different from zero to avoid singularities for any combination of the α-coefficients [7].

On the other hand, to implement the PI component of the proposed controller it is required to know the general form of the passive output $\tilde{y}$, which can be determined by remembering that it was defined as $\tilde{y}=g{\left(x^{⋆}\right)}^{T}\tilde{x}$ as presented below.

(32) $$\begin{array}{c} \tilde{y}=\left(\alpha_{3}E+\alpha_{2}x_{2}^{⋆}\right)\left(x_{1}-x_{1}^{⋆}\right)-\alpha_{2}x_{1}^{⋆}\left(x_{2}-x_{2}^{⋆}\right). \end{array}$$

Note that in the passivity-based control theory, the passive output, i.e., $\tilde{y}$, can be interpreted as a rate of power change, which goes to zero when the system reaches the assignable equilibrium point [34].

## 4. Sensorless-Based Estimator Applied Unknown Resistive Load

The main characteristic of the proposed PI passivity-based controller is the dependence of the control law *u* and the non-controlled current reference of the load value, i.e., the value of the $G_{L}$. However, in a real application to know the value of the load is not practical since this can variate as a function of the circuit requirements [21]. A classical approach to estimate the value of the load current is to use a current sensor in the load side which, in conjunction with the voltage sensor, it allows to determine the value of the resistance of the load by using Ohm’s law [39]. However, there exists a practical approach reported in [40] where it is possible to eliminate the load current sensor through the estimation of the load using only the voltage measurement. Here, we present the general formulation of this load estimator. For this purpose, let us define the estimation error ${\tilde{G}}_{L}$ as follows:

(33) $$\begin{array}{c} {\tilde{G}}_{L}={\hat{G}}_{L}-G_{L}, \end{array}$$

where ${\hat{G}}_{L}$ represents the estimated value for the conductance connected at the load side of the converter. The estimated load variable can be defined as a function of the measured voltage in the terminals of the capacitor as follows:

(34) $$\begin{array}{c} {\hat{G}}_{L}=\beta +C\gamma \alpha \left(x_{2}\right), \end{array}$$

where γ is a positive constant, and the parameter β and the function $\alpha \left(x_{2}\right)$ must be designed to guarantee the exponential convergence of the load estimator [40].

Now, taking the time derivative of the estimation error, the following result is reached:

(35) $$\begin{array}{cc} {\dot{\tilde{G}}}_{L} & ={\dot{\hat{G}}}_{L},\\ \; & =\dot{\beta }+C\gamma \alpha^{\prime }\left(x_{2}\right){\dot{x}}_{2},\\ \; & =\dot{\beta }+C\gamma \alpha^{\prime }\left(x_{2}\right)\left(\frac{\alpha_{1}}{C}x_{1}-\frac{G_{L}}{C}x_{2}-\frac{\alpha_{2}}{C}ux_{1}\right),\\ \; & =\dot{\beta }+\gamma \alpha^{\prime }\left(x_{2}\right)\left(\alpha_{1}x_{1}-G_{L}x_{2}-\alpha_{2}ux_{1}\right). \end{array}$$

To relate the derivative of the estimation error, its own variable from (33) is substituted $G_{L}$ in (35), which produces:

(36) $$\begin{array}{c} {\dot{\tilde{G}}}_{L}=\dot{\beta }+\gamma \alpha^{\prime }\left(x_{2}\right)\left(\alpha_{1}x_{1}-\left({\hat{G}}_{L}-{\tilde{G}}_{L}\right)x_{2}-\alpha_{2}ux_{1}\right). \end{array}$$

From (36) it is possible to obtain a general form for the time derivative of β, which can be assigned as follows [7]:

(37) $$\begin{array}{c} \dot{\beta }=-\gamma \alpha^{\prime }\left(x_{2}\right)\left(\alpha_{1}x_{1}-{\hat{G}}_{L}x_{2}-\alpha_{2}ux_{1}\right). \end{array}$$

Now, if we substitute (37) in (36), then, the following expression yields:

(38) $$\begin{array}{c} {\dot{\tilde{G}}}_{L}=\gamma \alpha^{\prime }\left(x_{2}\right){\tilde{G}}_{L}x_{2}. \end{array}$$

To ensure the exponential convergence of the load estimator, we proceed to the define the structure of the function $\alpha \left(x_{2}\right)$ as follows:

(39) $$\begin{array}{c} \alpha \left(x_{2}\right)=-\frac{1}{2}x_{2}^{2}, \end{array}$$

which can be derived with respect to $x_{2}$ and substituted in (38) producing the following result:

(40) $$\begin{array}{c} {\dot{\tilde{G}}}_{L}=-\gamma {\tilde{G}}_{L}x_{2}^{2}, \end{array}$$

(41) $$\begin{array}{c} {\tilde{G}}_{L}={\tilde{G}}_{L}\left(0\right)\exp^{\left(-\gamma x_{2}^{2}t\right)}, \end{array}$$

Note that the solution of (40) in (41) shows that the error of the load estimator goes to zero for any initial condition ${\tilde{G}}_{L}\left(0\right)$ with exponential convergence, which demonstrates that the value of ${\hat{G}}_{L}$ is equal to the real load $G_{L}$. This is important since in the control law (29) and the current reference (31), it is possible to substitute the real $G_{L}$ for the estimated value ${\hat{G}}_{L}$, maintaining the asymptotic convergence of the proposed PI passivity-based controller for regulating voltage in second-order DC-DC converters without using current load sensors.

## 5. Simulation and Experimental Results

**Figure 2:**
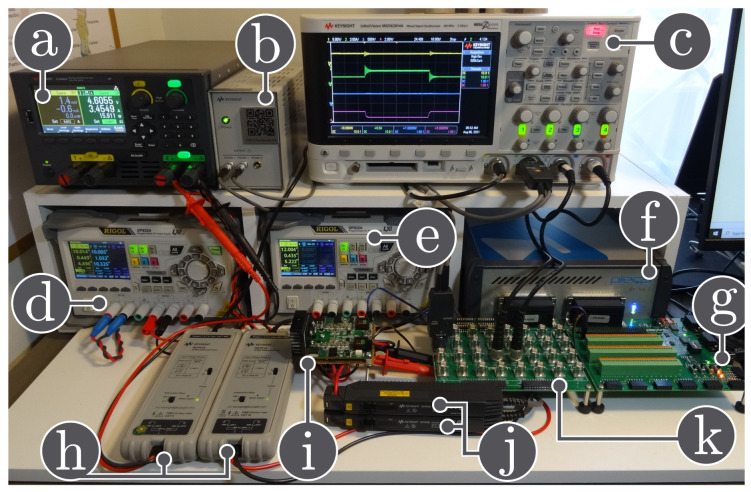
Experimental setup: (**a**) dc electronic load in constant resistance mode, (**b**) power supply for the current probes, (**c**) oscilloscope, (**d**) input dc power supply, (**e**) auxiliary power supply, (**f**) PLECS RT-box, (**g**) digital breakout board, (**h**) voltage differential probes, (**i**) reconfigurable power converter, (**j**) current probes, (**k**) analog breakout board.

This section presents the performance of the adaptive generalized PI-PBC applied to DC-DC converters feeding unknown loads in order to regulate the output voltage. Simulations and experimental results are used to assess the performance of the proposed controller. The simulations are performed in PLECs software, while the PLECS RT-box controls the converter prototypes as shown in Figure 2 and their components are listed in Table 2. In addition, the proposed controller is also compared to the conventional PI controller.

**Figure 3:**
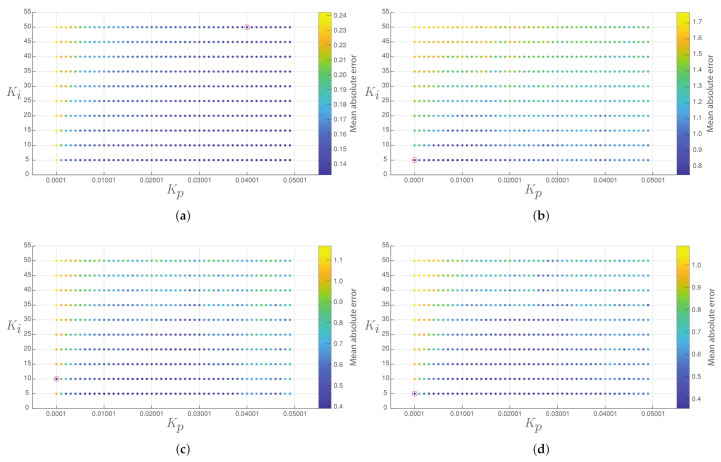
Mean absolute error of the output voltage in each converter (the best response in the red circle): (**a**) Buck converter, (**b**) Boost converter, (**c**) Buck-boost converter, and (**d**) Non-inverting buck-boost converter.

The tuning of the conventional PI controller for each converter is performed through a systematic sweep that has considered 500 different configurations of the constants $K_{p}$ and $K_{i}$. Figure 3 presents the mean absolute error of the output voltage in each converter for the different values of control gains studied, and Table 3 shows the selected constants.

**Figure 4:**
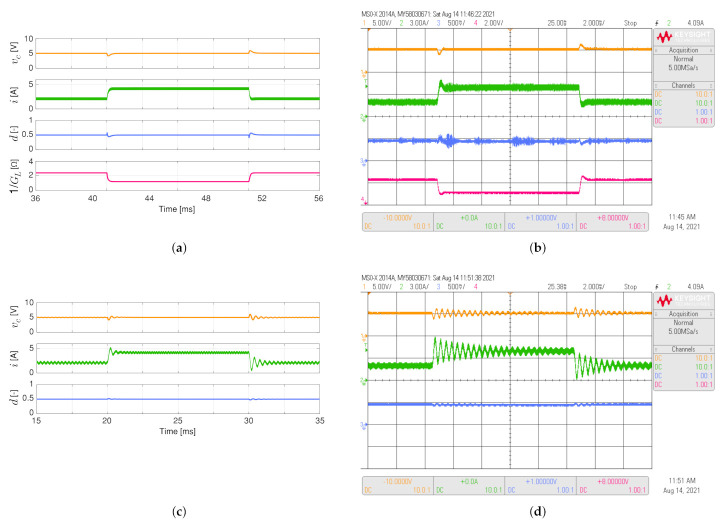
Simulated (**a**,**c**) and experimental (**b**,**d**) dynamic responses of the buck converter when the load resistance is a 50 Hz square waveform between 1.2 Ω and 2.4 Ω, and a duty cycle of 0.5: (**a**,**b**) adaptive PI-PBC controller, (**c**,**d**) PI controller. CH1: $v_{c}$ (5 V/div), CH2: *i* (3 A/div), CH3: *d* (500 mV/div), CH4: $1/G_{L}$ ( 2 V/div) (it is calculated only by the PI-PBC controller), and time base of 2 ms.

Figure 4 shows the simulated and experimental responses of the buck converter considering the load resistance varies between 1.2 Ω and 2.4 Ω like a 50 Hz square waveform. For this converter, its control objective is to maintain the output voltage at 5 V. The proposed adaptive controller has a better dynamic response than the conventional PI controller since the settling times ($T_{s}$), when the load resistor changes, are shorter for the PI-PBC ($T_{s}$ about to 1.5 ms) than the conventional PI controller ($T_{s}$ about to 16 ms). In addition, the inductor current presents lower overshoots when the load resistor changes.

**Figure 5:**
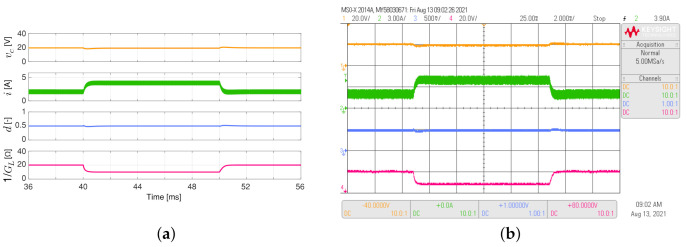

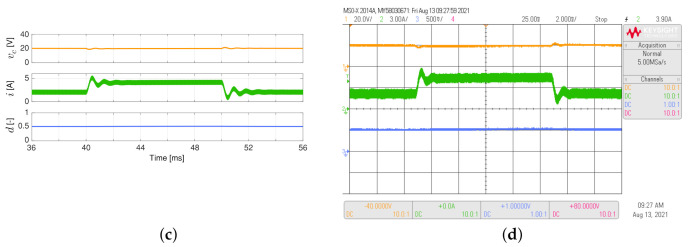
Simulated (**a**,**c**) and experimental (**b**,**d**) dynamic responses of the boost converter when the load resistance is a 50 Hz square waveform between 10 Ω and 20 Ω, and a duty cycle of 0.5: (**a**,**b**) adaptive PI-PBC controller, (**c**,**d**) PI controller. CH1: $v_{c}$ (20 V/div), CH2: *i* (3 A/div), CH3: *d* (500 mV/div), CH4: $1/G_{L}$ (20 V/div) (it is calculated only by the PI-PBC controller), and time base of 2ms.

The simulated and experimental responses of the boost converter are illustrated in Figure 5. This converter regulates its output voltage at 20 V under load resistance changes between 10 Ω and 20 Ω in 50 Hz square waveform. The adaptive PI-PBC controller continues to perform better than the conventional PI controller. This is supported by the settling times for the output voltage and the overshoots for the inductor current, which are lower for the proposed controller ($T_{s}$ about to 1.0 ms for the proposed controller while $T_{s}$ about to 4.0 ms for the PI controller). Furthermore, the responses for the adaptive PI-PBC controller behave as a first-order system, indicating that its responses are not overshot.

**Figure 6:**
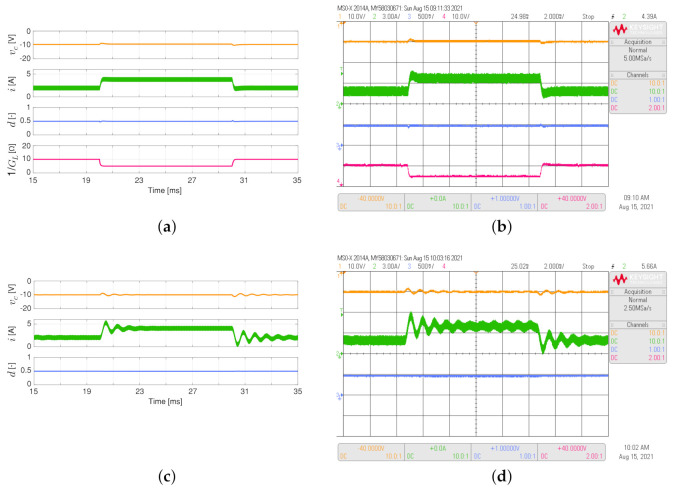
Simulated (**a**,**c**) and experimental (**b**,**d**) dynamic responses of the buck-boost converter when the load resistance is a 50 Hz square waveform between 5 Ω and 10 Ω, and a duty cycle of 0.5: (**a**,**b**) adaptive PI-PBC controller, (**c**,**d**) PI controller. CH1: $v_{c}$ (20 V/div), CH2: *i* (3 A/div), CH3: *d* (500 mV/div), CH4: $1/G_{L}$ ( 10 V/div) (it is calculated only by the PI-PBC controller), and time base of 2 ms.

Figure 6 depicts the simulated and experimental responses of the buck-boost converter feeding the load resistance, which varies between 5 Ω and 10 Ω as a 50 Hz square waveform with a duty cycle of 0.5. The adaptive proposed controller has better performance than the conventional PI controller. This is validated by comparing the settling times for the output voltage, which is lower for the adaptive PI-PBC controller ($T_{s}$ about to 1.2 ms for the proposed controller while $T_{s}$ about to 20.0 ms for the PI controller). At the same time, the inductor current overshoot for the proposed controller is lower without oscillations than the proposed controller (see the green line in Figure 6b,d).

**Figure 7:**
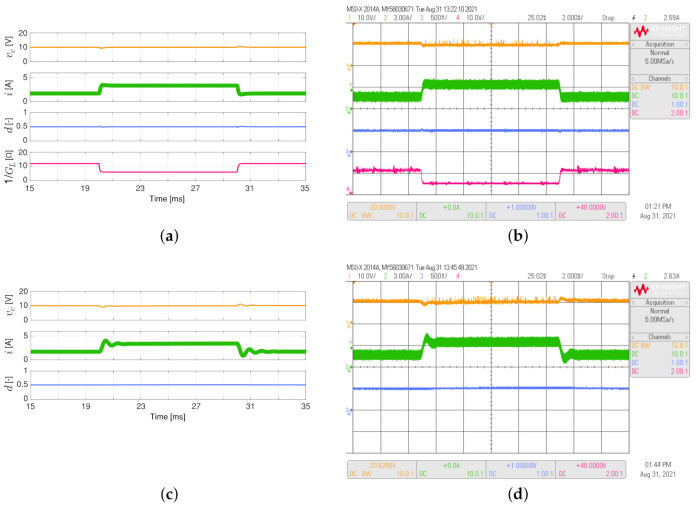
Simulated (**a**,**c**) and experimental (**b**,**d**) dynamic responses of the non-inverting buck-boost converter when the load resistance is a 50 Hz square waveform between 6 Ω and 12 Ω, and a duty cycle of 0.5: (**a**,**b**) adaptive PI-PBC controller, (**c**,**d**) PI controller. CH1: $v_{c}$ (10 V/div), CH2: *i* (3 A/div), CH3: *d* (500 mV/div), CH4: $1/G_{L}$ ( 10 V/div) (it is calculated only by the PI-PBC controller), and time base of 2 ms.

The simulated and experimental responses of the non-inverting buck-boost converter feeding the load resistance, which varies between 6 Ω and 12 Ω as a 50 Hz square waveform with a duty cycle of 0.5, are shown in Figure 7. The output voltage regulation for a non-inverting buck-boost converter is better when the adaptive controller is implemented. This can be supported by comparing the settling times in the experimental responses ($T_{s}$ about to 0.5 ms for the proposed controller while $T_{s}$ about to 2.5 ms for the PI controller, see Figure 7b,d). In addition, comparing the experimental responses for the inductor current, the proposed controller continues to present a better performance since there is no overshoot in this converter and its behavior is a first-order system.

According to the results shown, the proposed adaptive controller presented a better performance guaranteeing the system’s stability than the PI controllers. However, it is essential to mention the main disadvantages of the proposed controller, which are: the performance of the proposed controller depends on the proper modeling of the system; it is necessary to know all the variables and parameters of the system such as $v_{dc}$, *i*, and $R_{L}$; and it is only applicable to converters supplying resistive loads.

## 6. Conclusions

The classical DC-DC converters have modeled through a general bilinear representation using a port-Controlled Hamiltonian, which allowed a general PI-PBC controller design that ensured that the closed-loop operation for all the second-order DC-DC converters is stable in the sense of Lyapunov. An adaptive load estimator was employed to reduce the number of sensors to determine the value of the resistance connected at the converter terminals, with the main advantage that exponential convergence is ensured. This allows classified the proposed PI-PBC controller as an adaptive control methodology. Numerical results demonstrated that the proposed controller presented a voltage regulation output in all the converters with a like first-order behavior, which implied that under load variations, overshoots are not reported, which was not the case of the classical PI controllers where multiple oscillations appeared in the regulated voltage output. The efficiency of the PI-PBC approach was demonstrated in the settling time, which was at least four times faster than the classical PI approach in all the converter topologies.

Future work will study a general port-Controlled Hamiltonian model for all the studied topologies considering unknown constant power terminals that allow the application of the interconnection and damping assignment PBC design to obtain a general control law ensuring a control law closed-loop stability in the sense of Lyapunov.

## Figures and Tables

**Table 1:** α-coefficients for each one of the studied converters.

Converter	$\alpha_{1}$	$\alpha_{2}$	$\alpha_{3}$	$\alpha_{4}$
Buck	1	0	1	0
Boost	1	1	0	1
Buck-boost	−1	−1	1	0
Non-inverting buck-boost	1	1	1	0

**Table 2:** Components description of the reconfigurable power converter.

Component/Element	Description	Type/Value
*E*	Input voltage	10 V
$Q_{1}$ and $Q_{2}$	Power MOSFET	IRFB4510PBF
$D_{1}$ and $D_{2}$	Schottky Power Diode	MBR60H100CTG
*L*	Inductor	Wurth Elektronik 74435584700, 47 μH
*C*	Multilayer Ceramic Capacitor	TDK C5750X7S2A106M230KB, 10×10μF

**Table 3:** Selected PI constants for each one of the studied converters.

Converter	$K_{p}$	$K_{i}$
Buck	0.04	50.0
Boost	0.0001	5.0
Buck-boost	0.0001	10.0
Non-inverting buck-boost	0.0001	5.0

## Data Availability

No new data were created or analyzed in this study. Data sharing is not applicable to this article.
